# Historical migration and taxonomic entity of Korean endemic shrub *Lespedeza maritima* (Fabaceae) based on microsatellite loci

**DOI:** 10.1093/aobpla/plab009

**Published:** 2021-02-18

**Authors:** Dong-Pil Jin, Jong-Soo Park, Byoung-Hee Choi

**Affiliations:** Department of Biological Sciences, Inha University, 100, Inha-ro, Michuhol-gu, Incheon 22212, Republic of Korea

**Keywords:** Bayesian clustering analysis, ecological niche modelling, establishment history, genetic diversity, Korean endemic shrub, last glacial maximum, *Lespedeza maritima*, microsatellite, morphological examination, taxonomy

## Abstract

Various plant species are endemic to the Korean Peninsula, but their evolutionary divergence and establishment are poorly understood. One of these, *Lespedeza maritima*, has been proposed as either a hybrid (*L*. *cyrtobotrya* × *L*. *maximowiczii*) or a synonym of *L*. *thunbergii*. A distinct taxon, *L*. *uekii*, has been proposed for inland populations. We investigated genetic diversity and structure in *L*. *maritima* and related taxa to resolve this. Genotypes of *L*. *maritima* (*n* = 244, including *L*. *uekii*) were determined using 12 microsatellite loci, then compared with those of related species. Genetic diversity within *L*. *maritima* was estimated, and Bayesian clustering analysis was used to represent its genetic structure and that of related taxa. Its distribution during the last glacial maximum (LGM) was predicted using ecological niche modelling (ENM). Neighbour-joining (NJ) analysis and principal coordinate analysis (PCoA) were used to investigate relationships among species. Bayesian tree based on nuclear ribosomal internal transcribed spacers (nrITS) was also reconstructed to show relationships and divergence time among species. Morphological features were examined using flower characteristics. In result, expected heterozygosity (*H*_E_) and allelic richness (*A*_R_) within *L*. *maritima* were higher in southern than northern populations. Bayesian clustering analysis largely assigned populations to two clusters (*K* = 2) (south vs. north). The ENM showed that *L*. *maritima* occurred around the East China Sea and Korean Strait land bridge during the LGM. Compared with other *Lespedeza* species, *L*. *maritima* was assigned to an independent cluster (*K* = 2–5), supported by the NJ, PCoA, Bayesian tree and morphological examination results. *Lespedeza maritima* and *L*. *uekii* were clustered to one clade on Bayesian tree. Given results, current *L*. *maritima* populations derive from post-LGM colonization away from southern refugia. The type *L*. *uekii*, which grows inland, is thought synonym of *L*. *maritima*. In addition, *L*. *maritima* is considered a distinct species, compared with related taxa.

## Teaser

The establishment process of *Lespedeza maritima* is inferred in this study. The taxonomic entity of this species is also described with comparison of related species. The genetic diversity and structure of *L. maritima* based on microsatellite loci suggest that current populations derive from post-LGM colonization away from southern refugia. Ecological niche modelling supports this. Compared with other *Lespedeza* species, molecular analyses (PCoA, NJ and Bayesian tree) and morphological examination indicate that *L. maritima* is a distinct species and *L. uekii* should be regarded as a synonym. This study could help to improve our understanding about the evolution of Korean endemic species.

## Introduction

The warm temperate forests of East Asia host many endemic species, making the region a global hotspot of species diversity (Xie 1997; Qian and Ricklefs 2000; Milne and Abbott 2002; Qiu *et al.* 2011). Climatic oscillations and changes in geography associated with the Quaternary are generally considered to have been the main driver governing the region’s present plant distributions (Hewitt 2004). During the last few decades, many studies on the genetic diversity and structure of plant species have sought to check this suggestion around East China Sea (ECS) (Qiu *et al.* 2009; Xu *et al.* 2015; Tang *et al.* 2017; Qian *et al.* 2019). Such studies mainly have focused on Chinese or Japanese plants, or both. However, recently, the migration and establishment history studies in East Asia including Korean warm temperate forests have also increased (Lee *et al.* 2013, 2014; Chung *et al.* 2014, 2018; Jin *et al.* 2016b; Han *et al.* 2020). However, the evolutionary history of a species can also be shaped by species-specific factors, such as life cycle, pollinator relationships, dispersal ability and habitat ecology (Hewitt 1999; Qiu *et al.* 2011). It is perceived that more case studies are required to improve our understanding.

Approximately 360 plant species have been reported as endemic to the Korean Peninsula (Chung *et al.* 2017). During the Quaternary, the East Asian regions now comprising the Korean Peninsula, China and Japan were repeatedly connected via an emerged land bridge (Ota 1998; Kimura 2000), which would potentially have provided an opportunity for species dispersals. However, several plant species did not extend their range, possibly because of a limited dispersal ability, insufficient time to support migration due to recent diversification and an absence of suitable habitat. Therefore, we used an endemic plant as a study species to investigate the colonization patterns of plants endemic to the Korean Peninsula.

*Lespedeza maritima* (Fabaceae) is a Korean endemic shrub that is primarily distributed in warm temperate forests along the southern and eastern coasts of the Korean Peninsula (Lee 1965; Choi 2007); some populations are found in relatively inland mountainous areas (Lee 1965). It is distinguished from other species within the genus by the shiny and leathery upper surface of the leaflets (Lee 1965). Despite the distinctive morphological characters of this species, it poses complex problems in taxonomy. Shortly after the first description of *L*. *maritima* (Nakai 1923), *L*. *uekii*, which has a similar morphology, was described (Nakai 1928). *Lespedeza maritima* was first collected on Bogil island (Wando-gun, Jeollanam-do), at the western end of the South Sea of Korea, whereas *L*. *uekii* was described based on specimens sampled at Dongnae beach (Busan-si), which is situated at the opposite side of the South Sea. The former is smaller (ca. 0.4 m) than the latter (up to 2 m), and its upper parts are more branched (Nakai 1928). Leaflets of *L*. *maritima* are generally ovate and obtuse at their apex, in comparison with the usually lanceolate and acute shape of the apex in *L*. *uekii* (Nakai 1923, 1928). Lee (1965) recognized *L*. *maritima* (including *L*. *uekii*) as a hybrid of *L*. *cyrtobotrya* and *L*. *maximowiczii*; the author furthermore suggested that *L*. *maritima* is closer to *L*. *cyrtobotrya* while *L*. *uekii* is closer to *L*. *maximowiczii*, based on morphology. In contrast, Akiyama (1988) treated the species as a synonym of *L*. *formosa* subsp. *velutina* [= *L*. *thunbergii* subsp. *thunbergii* (Ohashi *et al.* 2009)]. To date, no study has sought to resolve the taxonomic debate surrounding this species using the genetic method. In the present study, we therefore approached the issue by determining the genetic diversity and structure of the species and related taxa.

Our method involved the selection of previously developed microsatellite markers, to elucidate the phylogeographic history and taxonomic entity of the species (Jin *et al.* 2016a). Microsatellite loci are co-dominant and highly polymorphic (Duminil *et al.* 2012), and can be used for fine-scale evaluation of gene flows among populations and species genetic diversity (Sunnucks 2000). They are therefore a useful tool in research concerning phylogeography and population genetics (Sunnucks 2000; Lee *et al.* 2014). In addition, the detection of genetic alleles enables researchers to infer the establishment history of populations based on admixed lineages (Pritchard *et al.* 2000). Also, we applied a polymerase chain reaction (PCR) methodology to the nuclear ribosomal internal transcribed spacers (nrITS), because this is an appropriate method to elucidate the phylogeographic history determined in previous studies, and to enable the species divergence time to be calculated (Jin *et al.* 2016b).

Our overall study objectives were to (i) reveal the genetic diversity and structure of *L*. *maritima*, (ii) infer *L*. *maritima* refugia during the last glacial maximum (LGM) (~21 000 years ago) and the establishment process from that time and (iii) determine the appropriate taxonomic classification of *L*. *maritima* by comparing its genetic features with those of related taxa. Our research provides a case study that contributes to revealing the evolutionary establishment of Korean warm temperate plants, and the taxonomy of the *Lespedeza* genus.

## Materials and Methods

### Sampling

**Figure 1.**
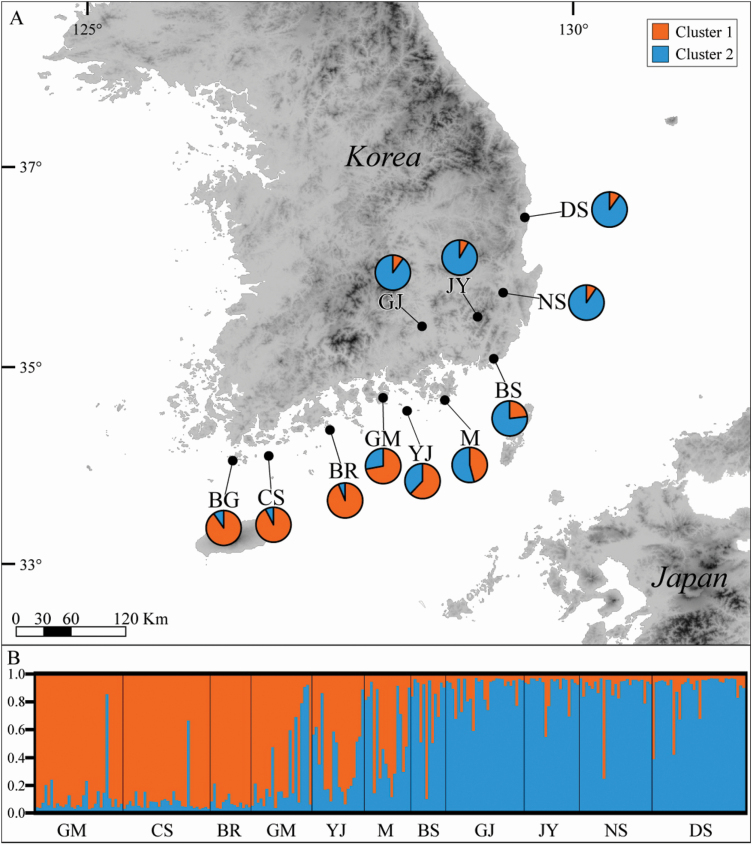
Sampling locations and genetic structure of *Lespedeza maritima*, determined using Bayesian clustering analysis. The ancestral clusters are distinguished according to their colour: cluster 1, orange; cluster 2, blue. (A) Sampling sites and composition of ancestral clusters of *L*. *maritima* populations. (B) The result of Bayesian clustering analysis for *L*. *maritima* based on 12 microsatellite loci.

We sampled 244 individuals of *L*. *maritima*  **[see**  **Supporting Information—Fig. S1****]** from 11 populations of Korea, to represent entire distribution (Fig. 1A; Table 1). In this sampling, we included individuals that were regarded as type *L*. *uekii*, found in mainly inland populations (BS, GJ, JY, NS and DS). Each populations included 12–32 individuals. In addition, we collected samples of *L*. *cyrtobotrya* (*n* = 42), *L*. *maximowiczii* (*n* = 41) and *L*. *thunbergii* (*n* = 22) to investigate the relationship with *L*. *maritima*. The sampling information of related species is provided in **Supporting Information—Table S1**. All samples were taken from adult plants spaced at least 5 m apart to avoid duplication. Leaf tissues were dried with silica-gel for DNA extraction. Voucher specimens were deposited in the Herbarium of Inha University (IUI).

**Table 1.** Locality and estimated genetic diversity parameters of *Lespedeza maritima* based on 12 microsatellite loci. *N*, number of samples; *N*_A_, observed number of alleles; *A*_R_, mean of allelic richness based on 14 samples; *H*_O_, observed heterozygosity; *H*_E_, expected heterozygosity; *F*_IS_, inbreeding coefficient.

Locality	Coordinate	Code	*N*	*N*_A_	*A*_R_	*H*_O_	*H*_E_	*F*_IS_
Southern group (island)								
Isl. Bogildo, Wando, Jeonnam	34°8′16″N/126°32′8″E	BG	30	30	4.643	0.556	0.611	0.090
Isl. Cheongsando, Wando, Jeonnam	34°11′42″N/126°53′43″E	CS	30	30	5.646	0.461	0.647	0.321
Isl. Oenarodo, Goheung, Jeonnam	34°26′16″N/127°30′20″E	BR	14	14	4.986	0.542	0.608	0.089
Mt. Gamabong, Namhae, Gyeongnam	34°44′51″N/128°1′7.62″E	GM	21	21	4.600	0.484	0.583	0.158
Isl. Yokjido, Tongyeong, Gyeongnam	34°38′15″N/128°16′11″E	YJ	18	18	4.693	0.505	0.584	0.178
Mt. Mangsan, Geoje, Gyeongnam	34°42′42″N/128°36′13″E	M	16	16	4.668	0.505	0.560^NS^	0.113
Region mean					4.873	0.509	0.599	0.158
Northern group (inland)								
Mt. Jangsangbong, Busan	35°6′10″N/129°6′13″E	BS	12	12	4.583	0.507	0.562^NS^	0.079
Mt. Gujinsan, Changnyeong, Gyeongnam	35°27′12″N/128°24′44″E	GJ	27	27	3.900	0.460	0.512	0.143
Mt. Jaeyaksan, Milyang, Gyeongnam	35°32′28″N/128°57′42″E	JY	19	19	4.090	0.513	0.534^NS^	0.041
Mt. Namsan, Gyeongju, Gyeongbuk	35°45′36″N/129°12′34″E	NS	25	25	4.343	0.513	0.529	0.019
Mt. Daesosan, Yeongdeok, Gyeongbuk	36°30′26″N/129°25′35″E	DS	32	32	3.607	0.404	0.466	0.129
Region mean					4.105	0.479	0.521	0.082
Total mean			244	244	4.524	0.495	0.563	0.124

### DNA extraction and PCR

Total genomic DNA was extracted from the silica-dried leaves of individuals with a MG Plant Genomic DNA Extraction SV miniprep (MGmed, Seoul, Korea). The process was as per the manufacturer’s manual. We used PCR amplification for 12 microsatellite loci and internal transcribed spacer of nuclear ribosomal DNA (nrITS), and the primers were employed as follows: LMS11, LMS18, LMS28, LMS33, LMS34, LMS39, LMS47, LMS53, LMS55, LMS58, LMS61 and LMS62 (Jin *et al.* 2016a). Those markers were selected by considering scoring results; they showed proper length variations according to repeat motif in GENEMAPPER 3.7 (Applied Biosystems, Foster City, CA, USA) and well-amplified marker across individuals. For amplification of nrITS, ITS4 and ITS5 (White *et al*. 1990) were also chosen. We conducted PCR with a GeneAmp® PCR System 2700 Thermal Cycler (Applied Biosystems). Each reaction mixture for microsatellite marker (10 μL total volume) contained 5 ng of DNA plus 5 μL of MG 2× *Taq* MasterMix with Dye (MGmed). The mixtures also contained the appropriate 0.08 μM forward M13 (-21)-tagged primer, a 0.3 μM reverse primer and a 0.3 μM M13 (-21)-labelled fluorescent marker (6-FAM, VIC, NED, PET). The PCR reaction mixture for nrITS (50 μL total volume) included MG 2× *Taq* MasterMix with Dye (MGmed), 10 ng of DNA, 0.3 μM primers and distilled water. Conditions included initial denaturation at 94 °C for 3 min; then 30 cycles at 94 °C for 30 s, 53 °C for 45 s and 72 °C for 45 s (1 min for nrITS); with no final extension (7 min for nrITS). Afterward, the PCR products were visualized on 2 % agarose gels. Microsatellite products were resolved to genotype on an ABI 3730XL sequencer with GeneScan™ 500LIZ™ size standards (Applied Biosystems). The sizes of the alleles were determined with GENEMAPPER 3.7, and the genotype data are provided in **Supporting Information—Appendix S1**. While, nrITS sequences were treated with a MG PCR Purification kit (MGmed), and sequenced throughout MACROGEN (Macrogen Inc., Seoul, Korea). The sequences identified in this study were deposited in GenBank database (MT919326–MT919335).

### Microsatellite data analyses

We evaluated genetic diversity by single population, region and total study area, by calculating genetic diversity parameters such as the number of alleles (*N*_A_), observed heterozygosity (*H*_O_), expected heterozygosity (*H*_E_) and Wright’s inbreeding coefficient (*F*_IS_), using GenAlEx version 6.503 (Peakall and Smouse 2006). FSTAT version 2.9.4 (Goudet 2005) was used to estimate allelic richness (*A*_R_). Deviation from the Hardy–Weinberg equilibrium (HWE) and linkage disequilibrium (LD) for each loci within populations was verified using the Hardy–Weinberg exact probability test and LD options provided in GenePop version 4.7 (Rousset 2008) with 10 000 Markov Chain Monte Carlo (MCMC) batches, 1000 iterations per batch and dememorization number = 1000. The null allele frequency was verified using MICRO-CHECKER version 2.2 (Van Oosterhout *et al.* 2004). The final genotyping data were formulated by considering allele sizes and null alleles.

A Bayesian clustering method using STRUCTURE version 2.3.4 (Pritchard *et al.* 2000) was employed to analyse the genetic structure of *L*. *maritima* and compare it with its related taxa. The simulation was run 10 times per cluster value (*K* = 1–16), employing 300 000 MCMC sampling runs after a burn-in period of 30 000 iterations and an admixture model under an assumption of correlated allele frequencies. Clustering Markov Packager Across K (CLUMPAK) software (Kopelman *et al.* 2015) was used to summarize and build a representative stacked bar chart based on the results associated with each *K* value generated by each run. The optimum *K* value was estimated according to Evanno *et al.* (2005), using STRUCTURE HARVESTER version 0.6.94 (Earl and vonHoldt 2012), which are shown in **Supporting Information—Fig. S2**.

In order to test the presence of isolation by distance (IBD), we estimated the genetic (*F*_ST_/(1 − *F*_ST_)) and geographic distances (km) between all population pairs (Rousset 1997). Correlations between genetic and geographic distances were calculated using the Mantel test option provided in GenAlEx version 6.5, with an assessment of statistical significance based on 9999 permutations.

The reduction in past population size was determined using BOTTLENECK version 1.2.02 (Piry *et al.* 1999). The programme was run using a Wilcoxon signed rank test with two mutational models provided by the programme: the stepwise mutation model (SMM), and two-phase model (TPM). Two-phase model was applied with a 95 % SMM; the variance among multiple steps was set to 12. Genetic drift was detected using allele frequency distribution analysis. If no L-shaped curve was observed, deviation from mutation equilibrium was assumed; this was in accordance with the programme author’s recommendation for settings using fewer than 20 microsatellite loci (Piry *et al.* 1999).

A neighbour-joining (NJ) tree was constructed with 1000 resampling and bootstrap values per locus, using POPTREE 2 (Takezaki *et al.* 2010), to reveal relationships among species. A genetic distance matrix test based on Nei’s genetic distance (*D*_A_) (Nei *et al.* 1983) was used to depict the phylogenetic tree. A principal coordinate analysis (PCoA) of pairwise individual genetic distance matrices (pairwise Nei’s genetic distances) was also conducted using GenAlEx 6.5.

### Divergence time estimate for *L. maritima* and related taxa

In order to estimate the divergence time of *L. maritima*, we took a Bayesian approach to calculating divergence times among this species and related taxa, using BEAST version 2.5.0 (Bouckaert *et al.* 2019). Except for our nrITS data sequenced in the present study, other data of tribe Desmodieae species were acquired from the study of DNA barcode (Jin *et al.* 2019). The Tamura–Nei (TrN) model of nucleotide substitution and a heterogeneity model based on site gamma distribution were selected using the Akaike Information criterion (AIC) in jModelTest version 2.1.6 (Darriba *et al.* 2012). Four rate categories were used under an uncorrelated lognormal relaxed clock model. We selected the ‘coalescent with birth and death model’ to model the tree representing time prior to divergence. Four calibration points were selected based on previously reported estimated values (Lavin *et al.* 2005; Xu *et al.* 2012). These points were: 14.2 million years ago (mya) (95 % highest posterior density [HPD]: 10.7–17.7) at *Desmodium*/*Campylotropis*; 11.9 million mya (95 % HPD: 8.4–15.3) at the *Campylotropis*/*Kummerowia*-plus-*Lespedeza* node; 9.2 mya (95 % HPD: 6.3–14.4) at the *Kummerowia*/*Lespedeza* node; and 8.2 mya (95 % HPD: 5.4–11.1) at the American/Asian *Lespedeza* node. The BEAST analyses were performed with a burn-in period of 10 % generations, using data collected for a 10 000 000 MCMC cycle sampling. A total of 1 000 000 burn-in samples and 1000 iterations were included per MCMC cycle. The phylogenic evolutionary trees were confirmed used Figtree version 1.4.0 (http://tree.bio.ed.ac.uk/software/figtree/). The statistical values for the results parameters were observed using Tracer version 1.7 (Rambaut *et al.* 2018).

### Ecological niche modelling

Predictions were made of the current and LGM geographic distribution of suitable habitat for *L*. *maritima*, using Maxent version 3.4.1 (Merow *et al.* 2013). Actual current *L*. *maritima* locality data were acquired through field observations. The analysis followed the method of Park *et al.* (2019). Nineteen bioclimatic variables for the present and LGM were obtained from Climatologies at High Resolution for the Earth’s Land Surface Areas (CHELSA; http://chelsa-climate.org/; Karger *et al.* 2017). The elevation data during the LGM were also acquired from CHELSA; current elevation data (Global Multi-resolution Terrain Elevation Data; GMTED2010) (Danielson and Gesch 2011) were obtained from the USGS EROS Archive (https://www.usgs.gov/land-resources/eros/coastal-changes-and-impacts/gmted2010). Two models were used for past climate data relating to the LGM: the Community Climate System Model (CCSM4; Gent *et al.* 2011), and the Max Planck Institute for Meteorology Earth System Model (MPI-ESM-P). In order to avoid multicollinearity, we excluded the variables with a high Spearman correlation efficient (>0.9) by using SDMtoolbox 2.4 (Brown *et al.* 2017). After preliminary ecological niche modelling (ENM) run, variables with a contribution under 1 % were excepted. A final nine variables were included in predicting potential distribution; bio1 (annual mean temperature), bio2 (mean diurnal range), bio3 (isothermality), bio4 (temperature seasonality), bio5 (minimum temperature of coldest month), bio14 (precipitation in driest month), bio15 (precipitation seasonality), bio18 (precipitation in warmest quarter) and elevation. It is possible that *L*. *maritima* was distributed in China and/or Japan during the LGM, so we therefore extracted climate data for latitude 20–45°N and longitude 112–142°E using ArcGIS 10.5 (ERSI, Redlands, CA, USA). The following Maxent programme runs were performed with these settings: ‘create response curves’, ‘conduct jackknife tests’, ‘use 10 replicates of cross-validation’, ‘generate logistic output’, ‘select random seeds’ and ‘use 10 000 background points and 1000 iterations’.

### Morphological examination

We examined the morphological characters of *L. maritima* and related species (*L*. *cyrtobotrya*, *L*. *maximowcizii* and *L*. *thunbergii*) that are deposited in the Herbarium of Inha University (IUI). Additional morphological examination was conducted by observing specimen from the Korea National Arboretum (KH). All observations of morphological characters were made with a stereomicroscope (Leica MZ8; Wetzlar, Germany), and criteria for floral measurements were mainly those stipulated by Akiyama (1988).

## Results

### Genetic diversity and structure

The genetic diversity of *L. maritima* across populations was evaluated based on 12 microsatellite loci (Table 1). All the loci employed were polymorphic, therefore enabling the genetic diversity and structure of this species to be revealed. The *H*_E_ and *A*_R_ were variable; *H*_E_ within populations ranged from 0.466 (DS) to 0.647 (CS) and, based on 12 individuals, *A*_R_ was the highest in CS (*A*_R_ = 5.646), but the lowest in DS (*A*_R_ = 3.607). When comparing regions (south vs. north), south populations (*A*_R_ = 4.873; *H*_O_ = 0.509; *H*_E_ = 0.599) showed higher genetic diversity than north populations (*A*_R_ = 4.105; *H*_O_ = 0.479; *H*_E_ = 0.521). The inbreeding coefficient (*F*_IS_) varied from 0.019 (NS) to 0.321 (CS). Although *F*_IS_ was higher in CS than in other populations (mean, 0.124), *F*_IS_ values in the latter implied a low probability of inbreeding.

According to a Hardy–Weinberg exact test, M, BS and JY deviated from the HWE, but the other eight populations were in agreement with it (Table 1). At the level of loci, four (LMS47, LMS55, LMS58 and LMS61) deviated from the HWE (*P* > 0.05), but this was not the case for the other eight loci (*P* < 0.05). No LD was found in any of the locus pairs across all populations (*P* > 0.05). Using BOTTLENECK, all populations were shown to be at mutation-drift equilibrium on the basis of both TPM and SMM in a Wilcoxon’s test (*P* > 0.05) **[see**  **Supporting Information—Table S2****]**, i.e. there was no evidence for a recent bottleneck. The allele frequency distribution in all populations was represented by a normal L-shape, consistent with the results of the Wilcoxon’s test **[see**  **Supporting Information—Table S2****]**.

The genetic structure of *L. maritima* was determined using Bayesian clustering analysis (Fig. 1). The results were based on variable clusters (*K* = 1–16); the optimum cluster number was two (*K* = 2) **[see**  **Supporting Information—Fig. S2A****]**. Populations were mostly divided into two clusters by geographic region, i.e. south (from BG to BR; cluster 1) versus north (from GJ to DS; cluster 2) (Fig. 1). A further four populations were located at the contact area between the south and north regions and harboured an admixed lineage (Fig. 1). A strong and significant IBD pattern was detected in the correlations between genetic and geographic distance across all populations (*R*^2^ = 0.472; *P* < 0.001) **[see**  **Supporting Information—Fig. S3****]**.

### Ecological niche modelling

The operating characteristic curve for the *L*. *maritima* distribution modelling had a high value (AUC = 0.995; SD = 0.003), based on nine environmental variables. Among these variables, the predicted current distribution **[see**  **Supporting Information—Fig. S4****]** of *L*. *maritima* was sited along the southern coastline, including Jeju island, stretching towards a section of the eastern coastline, based on a high distribution suitability (>0.8). Although *L*. *maritima* is no longer found on Jeju island, this prediction was otherwise largely in agreement with the actual geographic distribution of *L*. *maritima*.

**Figure 2.**
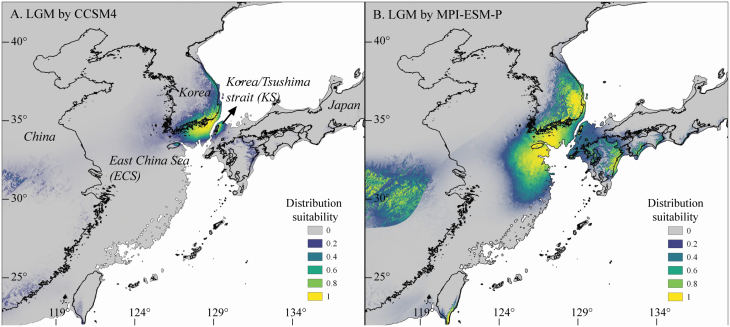
Potential distributions of *Lespedeza maritima* during the LGM, inferred using ENM and based on two climate models: (A) the Community Climate System Model (CCSM4); and (B) the Max Planck Institute for Meteorology Paleoclimate Model (MPI-ESM-P).

Two models used to predict the distribution of *L*. *maritima* during the LGM yielded significantly different results. Firstly, CCSM4 indicated that the area around the Korea/Tsushima Strait (KS) land bridge was a potential channel of *L*. *maritima* distribution (distribution suitability > 0.8) (Fig. 2A). In contrast, MPI-ESM-P suggested that a considerably wider area was suitable for *L*. *maritima*, including inland areas of the Korean Peninsula, Kyushu (Japan), and ECS (distribution suitability > 0.8) (Fig. 2B). This model also predicted a distribution in inland China and south of Taiwan.

### Divergence time estimation

**Figure 3.**
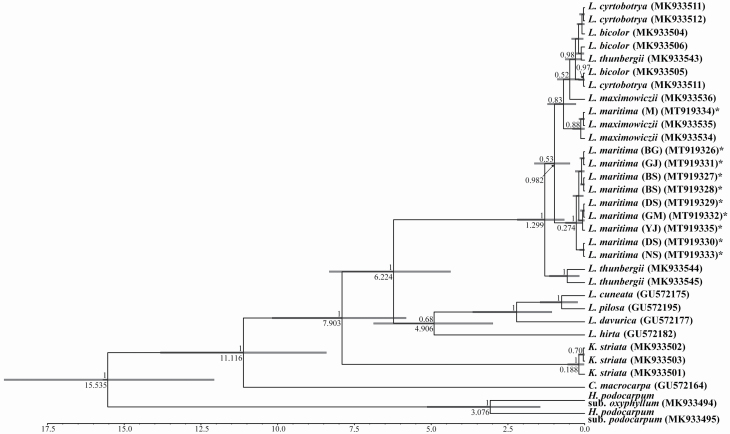
Estimates of divergence times for *Lespedeza maritima* and its related taxa using nrITS sequences, constructed using BEAST 2.5.0. The times and posterior values of the nodes are labelled to the side of and above the branch nodes, respectively. The greyish bar on the node represents the 95 % HPD age intervals. The bottom bar represents the timescale (mya from present). Newly sequenced individuals in this study are marked as asterisk. Population code of sequence in *L*. *maritima* is shown at side.

The nrITS sequences for *Lespedeza* species and other Desmodieae were aligned; the length was 656 bp. Effective sample sizes for each taxon group exceeded 1000, indicating that the chains were well-mixed. The mutation rate was estimated to be 0.689 × 10^–2^. Divergence times along the phylogenetic tree (Fig. 3) among genera (*Desmodium*, *Campylotropis*, *Kummerowia* and *Lespedeza*) were estimated as follows: 15.535 mya (95 % HPD: 12.068–18.912), *Hylodesmum* from other genera; 11.116 mya (95 % HPD: 8.409–13.822), *Campylotropis* from *Kummerowia*/*Lespedeza*; 7.903 mya (95 % HPD: 5.814–10.179), *Kummerowia* from *Lespedeza*; 6.224 mya (95 % HPD: 4.365–8.320), *Lespedeza* species; and 1.299 mya (95 % HPD: 0.661–2.197), *Macrolespedeza* from *Lespedeza*. Although most *Macrolespedeza* species were clustered polyphyletic, *L*. *maritima*, formed a clade excluding one individual (voucher no. 159039), close to *L*. *maximowiczii*. So, when this individual was discounted, the age the *L*. *maritima* node was the same as for *Lespedeza* species, i.e. 0.274 mya (95 % HPD: 0.207–1.579).

### Comparison of the genetic structure of *L. maritima* and its related taxa

**Figure 4.**
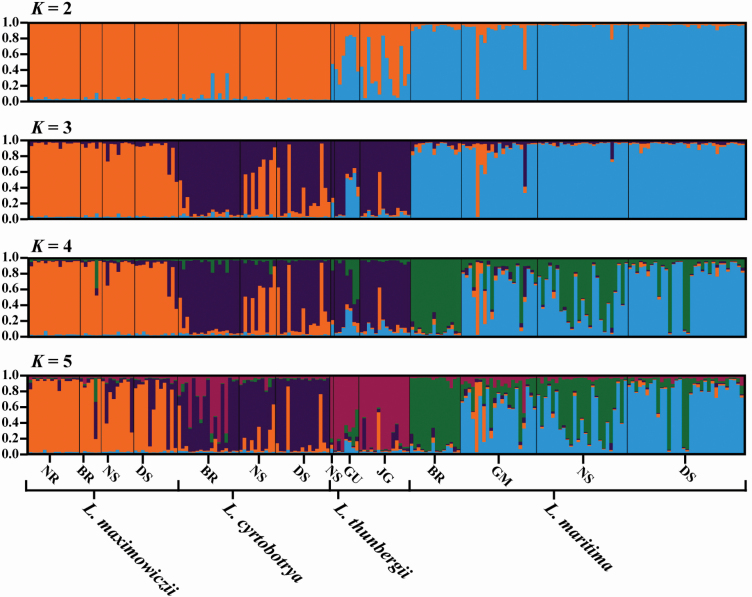
Genetic structure of *Lespedeza maritima* and related taxa, determined using Bayesian clustering analysis. The ancestral clusters are distinguished according to their colour: cluster 1, orange; cluster 2, blue; cluster 3, dark purple; cluster 4, green; cluster 5, reddish purple.

**Figure 5.**
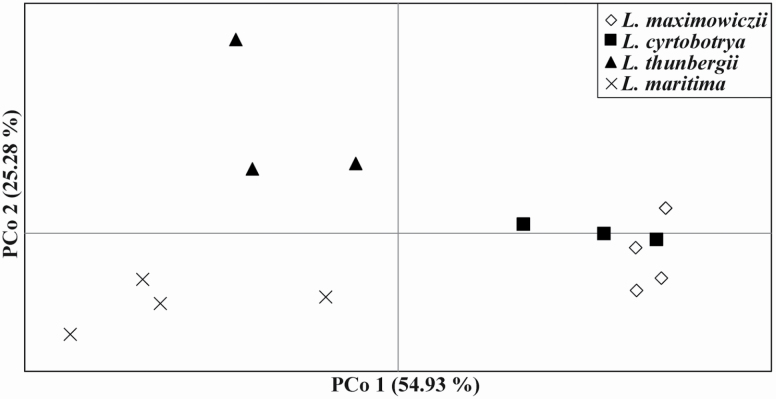
Principal coordinate analysis (PCoA) for *Lespedeza maritima* and its related taxa based on eight microsatellite loci. Species are distinguished by shape: diamond, *L. maximowiczii*; quadrangle, *L. cyrtobotrya*; triangle, *L*. *thunbergii* × *L*. *maritima*. Each dot corresponds to a population.

The genetic structures of *L. maritima* and three related species (*L*. *cyrtobotrya*, *L*. *maximowiczii* and *L*. *thunbergii*) were compared using Bayesian clustering analysis (Fig. 4); the optimum number of clusters was two (*K* = 2) **[see**  **Supporting Information—Fig. S2B****]**. *Lespedeza maritima* was assigned to cluster 2, while *L*. *cyrtobotrya* and *L*. *maximowiczii* were primarily assigned to cluster 1. Most *L*. *thunbergii* samples were showed an intermediate lineage, i.e. the posterior probability of a major cluster was below 0.8. Although ∆*K* values were low (since *K* = 3), more detailed divergences were observed among taxa. Divergence was found within *L*. *maritima* at *K* = 4 and 5 (i.e. BR [cluster 3] vs. GM, NS and DS [cluster 2]). *Lespedeza cyrtobotrya* (cluster 4) was separated from *L*. *maximowiczii* at *K* = 3. *Lespedeza thunbergii* could be distinguished from other taxa since *K* = 5 (cluster 5). The results of the PCoA determined genetic differences among the taxa, showing that *L*. *maritima* was distantly separated from other taxa (Fig. 5). Individuals in the other taxa were also clustered by taxon, though there was an overlap between part of *L*. *cyrtobotrya* and *L*. *maximowiczii*. The NJ tree **[see**  **Supporting Information—Fig. S5****]** also showed *L*. *maritima* to form independent clade from other taxa with high bootstrap value (84 %).

## Discussion

### Genetic diversity and inference of the glacial refugia of *L. maritima*

The results of the present study determined the genetic diversity and structure of *L. maritima*, a Korean endemic warm temperate shrub, across its distribution range (Table 1; Fig. 1). Based on microsatellite loci (*A*_R_ = 4.524, *H*_E_ = 0.563; Table 1), the species showed a moderate degree of genetic diversity in comparison with other warm temperate plants of East Asia, such as species of *Castanopsis* (Aoki *et al.* 2014), *Forsythia suspensa* (Fu *et al.* 2016), *Kirengeshoma palmata* (Yuan *et al.* 2014) and *Achyranthes bidentata* (Yan *et al.* 2016). More common and widespread species have a high genetic diversity in comparison with species endemic to a narrow region, which are prone to harbouring low genetic diversity, probably due to the influence of genetic drift or founder events (Frankham 1997; Cole 2003). *Lespedeza maritima* is narrowly distributed along the southern to eastern coastal line of the Korean Peninsula, so a low genetic diversity was expected. However, this species was predicted to have occurred over a wider area during the LGM compared with its current distribution (Fig. 2), and is not scarce within each of its current habitats. This previous wider range coupled with its abundance within currently occupied habitats could explain the moderate rather than low genetic diversity in *L*. *maritima*.

At the population level, the genetic diversity values determined for southern populations (*A*_R_ = 4.873, *H*_E_ = 0.599) were on average higher than those of northern populations (*A*_R_ = 4.105, *H*_E_ = 0.521) (Table 1). The ‘southern richness versus northern purity’ pattern of latitudinal variation in genetic diversity is commonly found in other temperate plants, suggesting a southward range contraction during glacial periods (Hewitt 2000; Hu *et al.* 2009). During glacial periods in East Asia, warm temperate plants generally retreated to southern refugia located on Jeju island in south Japan, and south China (Qiu *et al.* 2011; Lee *et al.* 2014; Tamaki *et al.* 2019). Our simulations of the past distribution of *L. maritima* based on CCSM and MPI-ESM-P largely supported this scenario (Fig. 2).

However, the models in our study revealed differences in the distribution range during the LGM; one model (CCSM) indicated that the only refugium was the KS land bridge, but the other scenario (MPI-ESM-P) predicted that the ECS land bridge should also be included (Fig. 2). Considering our genetic data, the BG, CS and BR populations (*A*_R_ = 4.643–5.646, *H*_E_ = 0.608–0.647), which were mainly allocated to cluster 1, harboured a higher genetic diversity than the other populations (Table 1) and had a distinct genetic structure (Fig. 1). Thus, we considered that the ECS land bridge was likely to have been one of the refugia.

However, the past occurrence of *L*. *maritima* in Kyushu (south Japan) and on Jeju island is confusing, due to its current absence (Choi 2007). This is possibly because of a failure to compete with broad evergreen species, which began to displace low altitude forests in both locations after the LGM (Chung 2007; Gotanda *et al.* 2008; Hase *et al.* 2012). As a result, some deciduous temperate plants were forced towards higher altitude areas within their habitat, with some failing to survive. Field observations have revealed that *L*. *maritima* is generally found at the foot to middle regions of mountains; although some populations have been observed close to mountain tops, in these cases the altitude was not high (below 500 m). It is therefore possible that *L*. *maritima* was unable to thrive and survive in the harsher conditions of higher altitude environments. We excluded the possibility of the existence of refugia in China and Taiwan on the basis of the dispersal range of *Lespedeza* species (Yokoyama *et al.* 2000). Consequently, we concluded that the range of *L*. *maritima* shifted southwards during the LGM, including to ECS, KS, Jeju and Kyushu.

### The establishment history of *L. maritima* including *L. uekii*

Current populations of *L. maritima* may represent post-LGM colonization from southern refugia around ECS and the KS land bridge (see above). This distribution shift was a gradual process rather than rapid expansion, a conclusion that is supported by significant evidence of IBD (Illera *et al.* 2019) **[see**  **Supporting Information—Fig. S2****]**. Populations located at both ends of the distribution (north or south; all except for the intermediate populations GM, YJ, M and BS) were each, respectively, assigned to two clusters using STRUCTURE, but the intermediate populations (GM, YJ, M and BS) harboured an admixed genetic structure (Fig. 1). This gradient change from south to north appears to reflect a gradual dispersal from southern refugia. The DS northern populations (*A*_R_ = 3.607, *H*_E_ = 0.466) and GJ (*A*_R_ = 3.900, *H*_E_ = 0.512) had the lowest genetic diversity (Table 1). A BOTTLENECK test revealed no sign of recent genetic drift in any of the populations (*P* > 0.05) **[see**  **Supporting Information—Table S2****]**, suggesting they had been established for a long time; this was also inferred from the IBD results.

Many warm temperate plants of the Korean Peninsula, especially broad evergreens, are commonly distributed from south to north along the western coastal line under the influence of the warm ocean currents (Lee and Im 2002). In contrast, *L*. *maritima* occurs from along the southern to eastern coastline. Taking note of this, we hypothesized why *L*. *maritima* occurs in this distribution pattern. During the LGM, *L*. *maritima* mainly occurred around the ECS and KS land bridge (see above). As the post-LGM climate warmed, sea level began to rise (Fairbanks 1989; Mix *et al.* 2001) and warm temperate plants migrated northwards to what is the current southern coastal region of the Korean Peninsula as the land bridge became submerged (Lee *et al.* 2014; Tamaki *et al.* 2019). *Lespedeza maritima* was also forced to migrate to the current islands and coastline of the southern Korean Peninsula. Its settlement inland could have been limited by the drier and colder climate of the inland compared with coastal regions and the decreased influence of sea breezes (Lee and Im 2002). However, south-eastern populations were able to adapt that environment, and this is difficult to explain. In morphological terms, northern populations (form, *L*. *uekii*) show different characteristics to southern populations (form, *L*. *maritima*); northern individuals are usually taller (up to 2 m) and with acute and narrow leaflets, compared with the height (ca. 40 cm) and obtuse leaflet shape of southern individuals (Lee 1965). *Lespedeza uekii* has been regarded as a distinct species on the basis of these morphological characters (Nakai 1928). In the present study, we examined the tendency of *L*. *uekii* to produce large-sized flowers (Table 2). However, populations intermediate between the north and south (GM, YJ, M and BS) showed a mixed lineage (Fig. 1), implying gene flow exchanges between *L*. *maritima* and *L*. *uekii*, supported by the overlapping morphological variations of the two forms (Table 2). In addition, no divergence between the two taxa was found on the nrITS-based Bayesian tree (Fig. 3). In field survey, *L*. *maritima* and *L*. *uekii* often grow within the same habitat. Thus, the evidence for regarding the latter as an independent species is inadequate. We therefore concluded that *L*. *uekii* is a synonym of *L*. *maritima*, following the taxonomic treatment of Lee (1965).

**Table 2.** Comparison of morphological traits among *Lespedeza maritima* and its related taxa. Number in parenthesis indicates mean value. All measured values are expressed in millimetres.

	*L. maritima* (type *L. maritima*)	*L. maritima* (type *L. uekii*)	*L. thunbergii*	*L. cyrtobotrya*	*L*. *maximowiczii*
Shape of bud	Terete	Terete	Terete	Terete	Flattened
Arrangement of bud scales	Spirally	Spirally	Spirally	Spirally	Mostly distichous
Length of inflorescence	Shorter than subtending leaf	Shorter than subtending leaf	Longer or slightly shorter than subtending leaf	Shorter than subtending leaf	Longer or slightly shorter than subtending leaf
Length of flower	11.9–(12.8)–13.4	14.0–(14.6)–15.7	11.0–(12.1)–13.0	9.0–(9.6)–10.3	8.2–(9.5)–10.9
Length of standard petal	10.3–(11.9)–13.2	11.9–(13.9)–15.0	9.5–(11.5)–12.3	8.8–(9.5)–10.2	7.0–(8.5)–10.0
Length of standard petal claw	1.6–(2.1)–2.5	2.1–(2.7)–3.1	1.6–(1.9)–2.2	1.6–(1.8)–1.9	0.7–(1.1)–1.5
Length of wing petal	8.2–(9.5)–10.6	8.8–(11.2)–12.5	8.2–(9.5)–10.6	8.4–(8.7)–9.0	6.4–(7.5)–9.2
Length of wing petal claw	2.3–(3.2)–3.7	3.3–(3.9)–4.4	2.2–(2.8)–3.4	2.9–(3.3)–3.6	1.7–(2.4)–2.9
Length of wing petal lamina	6.0–(6.5)–7.1	5.7–(7.3)–8.4	5.6–(6.8)–7.7	5.1–(5.3)–5.5	4.5–(5.4)–6.7
Length of keel petal	11.1–(11.8)–12.7	11.9–(13.2)–15.1	10.9–(11.6)–12.2	7.3–(7.7)–8.0	7.8–(8.8)–9.8
Length of keel petal claw	3.4–(3.8)–4.3	3.8–(4.3)–4.7	2.6–(3.2)–3.8	3.3–(3.6)–3.9	2.0–(2.6)–3.3
Length of keel petal lamina	7.7–(8.2)–8.9	8.3–(9.2)–10.8	7.9–(8.6)–9.5	4.3–(4.3)–4.4	5.6–(6.3)–7.0
Length of calyx tube	1.8–(2.4)–2.8	2.3–(2.6)–3.4	1.4–(1.9)–3.1	2.4–(2.5)–2.8	1.2–(1.5)–2.1
Length of calyx lobe	2.6–(3.3)–4.0	2.7–(3.5)–4.0	2.0–(2.5)–3.1	3.4–(3.5)–3.6	1.5–(2.3)–3.5

### Taxonomic classification of *L. maritima*

The taxonomic position of *L*. *maritima* has been the subject of debate (Nakai 1923; Lee 1965; Akiyama 1988). It was regarded as a hybrid of *L*. *maximowiczii* and *L*. *cyrtobotrya* (Lee 1965), but Akiyama (1988) treated *L*. *maritima* as a synonym of *L*. *thunbergii* subsp. *thunbergii* (= *L*. *formosa* subsp. *velutina*). So, we examined these taxonomic views.

Our Bayesian clustering results implied that *L*. *maritima* was not the result of hybridization between putative parents (*L*. *cyrtobotrya* and *L*. *maximowiczii*) (Fig. 3); if this had been the case, then *L*. *maritima* would be expected to show an admixed lineage between *L*. *cyrtobotrya* and *L*. *maximowiczii* (e.g. Haselhorst and Buerkle 2013; Castillo-Mendoza *et al.* 2019), which it did not. On the nrITS-based Bayesian tree, *L*. *maritima* formed a clade separate from other species, with only one sample being closely related to *L*. *maximowiczii* (MT919334). The shiny and thick leaflets and long flowers (ca. 12 mm) of *L*. *maritima* is also distinguishable from the putative parents (Table 2). Therefore, we rejected the hybridization hypothesis for *L*. *maritima* (Lee 1965). The taxonomic treatment suggesting *L*. *maritima* to be a synonym of *L*. *thunbergii* was also inadequately supported. Although *L*. *thunbergii* is difficult to determine relationship between *L*. *maritima* at *K* = 2, this was assigned to a different ancestor from *K* = 3 (Fig. 4). Furthermore, populations of *L*. *thunbergii* could be separated from *L*. *maritima* based on the PCoA results (Fig. 5); this is a similar result to the independent clade formed by the NJ tree **[see**  **Supporting Information—Fig. S4****]**. *Lespedeza maritima* and *L*. *thunbergii* flowers are larger (mean length, > 12 mm) than other species in section *Macrolespedeza* (Table 2), and bloom later in the season (mainly in September) (Choi 2007). However, *L*. *maritima* (shrub) is woodier than *L*. *thunbergii* (subshrub), and the leaflet characters of the two are clearly different (Lee 1965). Therefore, we considered *L*. *maritima* and *L*. *thunbergii* to be different taxa, and that *L*. *maritima* is an independent species.

On the Korean Peninsula, *L. thunbergii* mainly grows at the foot of mountains, forest roads and roadsides, where it is prone to anthropogenic disturbance. Although *L. maritima* is distributed only in southern regions of Korea, this species often occupies habitats that *L*. *thunbergii* may prefer. Given their similar niche, we hypothesized that *L*. *maritima* and *L*. *thunbergii* have a shared most recent common ancestor (MRCA) compared with other related species. This assumption is supported by the PCoA (Fig. 5), which showed that *L*. *maritima* are more closely related to *L*. *thunbergii* than to *L*. *maximowiczii* and *L*. *cyrtobotyra*. The time to the MRCA of *L*. *maritima* and *L*. *thunbergii* is dated to the Early Pleistocene across East Asia (1.299 mya). Subsequently, the sea level rise during the Early to Middle Pleistocene (1.0–0.2 mya) caused the land bridge to become submerged, therefore limiting interregional migrations (Kimura 2000). Based on the nrITS, it is possible that the crown age of *L*. *maritima* (0.274 mya; Fig. 3) is included within that period. Stronger evidence is required to support this, as we did not include all of the East Asian section *Macrolespedeza*, and the resolution among species on the nrITS phylogenetic tree was low (Fig. 3).

## Supporting Information

The following additional information is available in the online version of this article—

**Appendix S1.** The genotype data of *Lespedeza* species used in this study.

**Table S1.** Sampling sites of *Lespedeza* species used for comparing genetic structure.

**Table S2.** Results of BOTTLENECK testing of 11 populations of *Lespedeza maritima* based on a two-phase model (TPM) and stepwise mutation model (SMM).

**Figure S1.**  *Lespedeza maritima*. (A) Habit. (B) Upper surface of leaflet. (C) Inflorescence. (D) Flower.

**Figure S2.** Graph of Δ*K* (*y*-axis) according to number of clusters (*K*) (*x*-axis) as calculated based on Bayesian clustering analysis. (A) Result of analysis for 11 populations of *Lespedeza maritima* (Fig. 1). (B) Result of analysis for *L*. *maritima* and related taxa (Fig. 4).

**Figure S3.** Isolation by distance (IBD) based on relationships between pairwise genetic distance (*F*_ST_/[1 − *F*_ST_]) (*x*-axis) and geographic distance (km) (*y*-axis) of *Lespedeza maritima* populations. A positive correlation was significant between two distances (*R*^2^ = 0.472; *P* < 0.001).

**Figure S4.** Potential distributions of *Lespedeza maritima* during the current, inferred using ecological niche modelling.

**Figure S5.** Neighbour-joining (NJ) tree for *Lespedeza maritima* and its related based on eight microsatellite loci. One thousand bootstrap matrices of Nei’s genetic distance (*D*_A_) (Nei *et al.* 1983) were implemented. The number of each node indicates the bootstrap value (>50 %).

plab009_suppl_Supplementary_Materials

plab009_suppl_Supplementary_Appendix

## Data Availability

The genotype data of *Lespedeza* species used in this study are available as **Supporting Information**. The sequence data of *Lespedeza maritima* used in this study are available from NCBI (https://www.ncbi.nlm.nih.gov/). The GenBank accession numbers are shown in Fig. 3.

## Notes

**Evolution & Diversity. Chief Editor:** Jeremy Beaulieu

## References

[CIT0001] Akiyama S. 1988. A revision of the genus *Lespedeza* section *Macrolespedeza* (Leguminosae). The University Museum, The University of Tokyo, Bulletin 33:1–170.

[CIT0002] Aoki K, Ueno S, Kamijo T, Setoguchi H, Murakami N, Kato M, Tsumura Y. 2014. Genetic differentiation and genetic diversity of *Castanopsis* (Fagaceae), the dominant tree species in Japanese broadleaved evergreen forests, revealed by analysis of EST-associated microsatellites. PLoS One 9:e87429. 24498103 10.1371/journal.pone.0087429 PMC3907500

[CIT0003] Bouckaert R, Vaughan TG, Barido-Sottani J, Duchêne S, Fourment M, Gavryushkina A, Heled J, Jones G, Kühnert D, De Maio N, Matschiner M, Mendes FK, Müller NF, Ogilvie HA, du Plessis L, Popinga A, Rambaut A, Rasmussen D, Siveroni I, Suchard MA, Wu CH, Xie D, Zhang C, Stadler T, Drummond AJ. 2019. BEAST 2.5: an advanced software platform for Bayesian evolutionary analysis. PLoS Computational Biology 15:e1006650. 30958812 10.1371/journal.pcbi.1006650 PMC6472827

[CIT0004] Brown JL, Bennett JR, French CM. 2017. SDMtoolbox 2.0: the next generation Python-based GIS toolkit for landscape genetic, biogeographic and species distribution model analyses. PeerJ 5:e4095. 29230356 10.7717/peerj.4095 PMC5721907

[CIT0005] Castillo-Mendoza E, Salinas-Sánchez D, Valencia-Cuevas L, Zamilpa A, Tovar-Sánchez E. 2019. Natural hybridization among *Quercus glabrescens*, *Q*. *rugosa* and *Q*. *obtusata* (Fagaceae): microsatellites and secondary metabolites markers. Plant Biology 21:110–121. 30117248 10.1111/plb.12899

[CIT0006] Chung CH. 2007. Vegetation response to climate change on Jeju Island, South Korea, during the last deglaciation based on pollen record. Geosciences Journal 11:147–155.

[CIT0007] Chung GY, Chang KS, Chung JM, Choi HJ, Paik WK, Hyun JO. 2017. A checklist of endemic plants on the Korean Peninsula. Korean Journal of Plant Taxonomy 47:264–288.

[CIT0008] Chung MY, Le HTQ, Son S, Tian HZ, Chung MG. 2018. Genetic diversity of the extremely rare *Habenaria dentata* and the rare *Habenaria linearifolia* (Orchidaceae) in South Korea: implications for population history and conservation. Plant Ecology and Evolution 151:48–60.

[CIT0009] Chung MY, López-Pujol J, Chung MG. 2014. Genetic homogeneity between Korean and Japanese populations of the broad-leaved evergreen tree *Machilus thunbergii* (Lauraceae): a massive postglacial immigration through the Korea Strait or something else? Biochemical Systematics and Ecology 53:20–28.

[CIT0010] Cole C. 2003. Genetic variation in rare and common plants. Annual Review of Ecology, Evolution, and Systematics 34:213–237.

[CIT0011] Danielson JJ, Gesch DB. 2011. Global multi-resolution terrain elevation data 2010 (GMTED2010). Garretson: U.S. Geological Survey Open-File Report 2011-1073.

[CIT0012] Darriba D, Taboada GL, Doallo R, Posada D. 2012. jModelTest 2: more models, new heuristics and parallel computing. Nature Methods 9:772. 10.1038/nmeth.2109 PMC4594756 22847109

[CIT0014] Duminil J, Kenfack D, Viscosi V, Grumiau L, Hardy OJ. 2012. Testing species delimitation in sympatric species complexes: the case of an African tropical tree, *Carapa* spp. (Meliaceae). Molecular Phylogenetics and Evolution 62:275–285. 22019936 10.1016/j.ympev.2011.09.020

[CIT0015] Earl DA, vonHoldt BM. 2012. STRUCTURE HARVESTER: a website and program for visualizing STRUCTURE output and implementing the Evanno method. Conservation Genetics Resources 4:359–361.

[CIT0016] Evanno G, Regnaut S, Goudet J. 2005. Detecting the number of clusters of individuals using the software STRUCTURE: a simulation study. Molecular Ecology 14:2611–2620. 15969739 10.1111/j.1365-294X.2005.02553.x

[CIT0017] Fairbanks RG. 1989. A 17,000-year glacio-eustatic sea level record: influence of glacial melting rates on the Younger Dryas event and deep ocean circulation. Nature 342:637–647.

[CIT0018] Frankham R. 1997. Do island populations have less genetic variation than mainland populations? Heredity 78:311–327. 9119706 10.1038/hdy.1997.46

[CIT0019] Fu ZZ, Lei YK, Peng DD, Li Y. 2016. Population genetics of the widespread shrub *Forsythia suspensa* (Oleaceae) in warm-temperate China using microsatellite loci: implication for conservation. Plant Systematics and Evolution 302:1–9.

[CIT0020] Gent PR, Danabasoglu G, Donner LJ, Holland MM, Hunke EC, Jayne SR, Lawrence DM, Neale RB, Rasch PJ, Vertenstein M, Worley PH, Yang ZL, Zhang M. 2011. The community climate system model version 4. Journal of Climate 24:4973–4991.

[CIT0021] Gotanda K, Nakagawa T, Tarasov PE, Yasuda Y. 2008. Disturbed vegetation reconstruction using the biomization method from Japanese pollen data: modern and Late Quaternary samples. Quaternary International 184:56–74.

[CIT0022] Goudet J. 2005. FSTAT (version 2.9.4): a program to estimate and test gene diversities and fixation indices. http://www2.unil.ch/popgen/softwares/fstat.htm (26 August 2020).

[CIT0023] Han EK, Cho WB, ParK JS, Choi IS, Kwak M, Kim BY, Lee JH. 2020. A disjunctive marginal edge of evergreen broad-leaved oak (*Quercus gilva*) in East Asia: the high genetic distinctiveness and unusual diversity of Jeju island populations and insight into a massive, independent postglacial colonization. Genes 11:1114. 10.3390/genes11101114 PMC7598624 32977695

[CIT0024] Hase Y, Iwauchi A, Uchikoshiyama U, Noguchi E, Sasaki N. 2012. Vegetation changes after the late period of the Last Glacial Age based on pollen analysis of the northern area of Aso Caldera in central Kyushu, Southwest Japan. Quaternary International 254:107–117.

[CIT0025] Haselhorst MSH, Buerkle CA. 2013. Population genetic structure of *Picea engelmannii*, *P*. *glauca* and their previously unrecognized hybrids in the central Rocky Mountains. Tree Genetics & Genomes 9:669–681.

[CIT0026] Hewitt GM. 1999. Post-glacial re-colonization of European biota. Biological Journal of the Linnean Society 68:87–112.

[CIT0027] Hewitt G. 2000. The genetic legacy of the Quaternary ice ages. Nature 405:907–913. 10879524 10.1038/35016000

[CIT0028] Hewitt GM. 2004. Genetic consequences of climatic oscillations in the Quaternary. Philosophical Transactions of the Royal Society of London. Series B, Biological Sciences 359:183–195. 15101575 10.1098/rstb.2003.1388 PMC1693318

[CIT0029] Hu FS, Hampe A, Petit RJ. 2009. Paleoecology meets genetics: deciphering past vegetational dynamics. Frontiers in Ecology and the Environment 7:371–379.

[CIT0030] Illera JC, Arenas M, López-Sánchez CA, Obeso JR, Laiolo P. 2019. Gradual distance dispersal shapes the genetic structure in an alpine grasshopper. Genes 10:590. 10.3390/genes10080590 PMC6724060 31387238

[CIT0032] Jin DP, Cho WB, Choi IS, Choi BH. 2016a. Isolation and characterization of 28 microsatellite loci for a Korean endemic, *Lespedeza maritima* (Fabaceae). Applications in Plant Sciences 4:1500089. 10.3732/apps.1500089 PMC4716779 26819860

[CIT0033] Jin DP, Lee JH, Xu B, Choi BH. 2016b. Phylogeography of East Asian *Lespedeza buergeri* (Fabaceae) based on chloroplast and nuclear ribosomal DNA sequence variations. Journal of Plant Research 129:793–805. 27206725 10.1007/s10265-016-0831-2

[CIT0034] Jin DP, Park JW, Park JS, Choi BH. 2019. DNA barcode and phylogenetic study of the tribe Desmodieae (Fabaceae) in Korea. Korean Journal of Plant Taxonomy 49:224–239 (in Korean with English abastract).

[CIT0035] Karger DN, Conrad O, Böhner J, Kawohl T, Kreft H, Soria-Auza RW, Zimmermann NE, Linder HP, Kessler M. 2017. Climatologies at high resolution for the earth’s land surface areas. Scientific Data 4:170122. 28872642 10.1038/sdata.2017.122 PMC5584396

[CIT0036] Kimura M. 2000. Palegeography of the Ryukyu Island. Tropics 10:5–24.

[CIT0037] Kopelman NM, Mayzel J, Jakobsson M, Rosenberg NA, Mayrose I. 2015. Clumpak: a program for identifying clustering modes and packaging population structure inferences across K. Molecular Ecology Resources 15:1179–1191. 25684545 10.1111/1755-0998.12387 PMC4534335

[CIT0038] Lavin M, Herendeen PS, Wojciechowski MF. 2005. Evolutionary rates analysis of Leguminosae implicates a rapid diversification of lineages during the tertiary. Systematic Biology 54:575–594. 16085576 10.1080/10635150590947131

[CIT0039] Lee TB. 1965. The *Lespedeza* of Korea (1). Bulletin of the Seoul National University Forests 2:1–43.

[CIT0040] Lee WC, Im YJ. 2002. Plant geography with special references to Korea. Chuncheon: Kangwon National University Press (in Korean).

[CIT0041] Lee JH, Lee DH, Choi BH. 2013. Phylogeography and genetic diversity of East Asian *Neolitsea sericea* (Lauraceae) based on variations in chloroplast DNA sequences. Journal of Plant Research 126:193–202. 22990429 10.1007/s10265-012-0519-1

[CIT0042] Lee JH, Lee DH, Choi IS, Choi BH. 2014. Genetic diversity and historical migration patterns of an endemic evergreen oak, *Quercus acuta*, across Korea and Japan, inferred from nuclear microsatellites. Plant Systematics and Evolution 300:1913–1923.

[CIT0043] Merow C, Smith MJ, Silander JA Jr. 2013. A practical guide to MaxEnt for modeling species’ distributions: what it does, and why inputs and settings matter. Ecography 36:1058–1069.

[CIT0044] Milne RI, Abbott RJ. 2002. The origin and evolution of Tertiary relict floras. Advances in Botanical Research 38:281–314.

[CIT0045] Mix AC, Bard E, Schneider R. 2001. Environmental processes of the ice age: land, oceans, glaciers (EPILOG). Quaternary Science Reviews 20:627–657.

[CIT0046] Nakai T. 1923. Notulae ad plantas Japoniae et Coreae XXX. Botanical Magazine Tokyo 37:69–82.

[CIT0047] Nakai T. 1928. Notulae ad Plantas Japoniae et Koreae XXXVI. Botanical Magazine Tokyo 42:451–479.

[CIT0048] Nei M, Tajima F, Tateno Y. 1983. Accuracy of estimated phylogenetic trees from molecular data. Journal of Molecular Evolution 19:153–170. 6571220 10.1007/BF02300753

[CIT0049] Ohashi H, Nemoto T, Ohashi K. 2009. A revision of *Lespedeza* subgenus *Macrolespedeza* (Leguminosae) in China. Journal of Japanese Botany 84:197–223.

[CIT0050] Ota H. 1998. Geographic patterns of endemism and speciation in amphibians and reptiles of the Ryukyu Archipelago, Japan, with special reference to their paleogeographical implications. Researches on Population Ecology 40:189–204.

[CIT0051] Park JS, Takayama K, Suyama Y, Choi BH. 2019. Distinct phylogeographic structure of the halophyte *Suaeda malacosperma* (Chenopodiaceae/Amaranthaceae), endemic to Korea–Japan region, influenced by historical range shift dynamics. Plant Systematics and Evolution 305:193–203.

[CIT0052] Peakall R, Smouse PE. 2006. GENALEX 6: genetic analysis in Excel. Population genetic software for teaching and research. Molecular Ecology Notes 6:288–295. 10.1093/bioinformatics/bts460 PMC3463245 22820204

[CIT0053] Piry S, Luikart G, Cornuet JM. 1999. Bottleneck: a computer program for detecting recent reductions in the effective population size using allele frequency data. Journal of Heredity 90:502–503.

[CIT0054] Pritchard JK, Stephens M, Donnelly P. 2000. Inference of population structure using multilocus genotype data. Genetics 155:945–959. 10835412 10.1093/genetics/155.2.945 PMC1461096

[CIT0055] Qian ZH, Li Y, Li MW, He YX, Li JX, Ye XF. 2019. Molecular phylogeography analysis reveals population dynamics and genetic divergence of a widespread tree *Pterocarya stenoptera* in China. Frontiers in Genetics 10:1089. 31737056 10.3389/fgene.2019.01089 PMC6838215

[CIT0056] Qian H, Ricklefs RE. 2000. Large-scale processes and the Asian bias in species diversity of temperate plants. Nature 407:180–182. 11001054 10.1038/35025052

[CIT0057] Qiu YX, Fu CX, Comes HP. 2011. Plant molecular phylogeography in China and adjacent regions: tracing the genetic imprints of Quaternary climate and environmental change in the world’s most diverse temperate flora. Molecular Phylogenetics and Evolution 59:225–244. 21292014 10.1016/j.ympev.2011.01.012

[CIT0058] Qiu YX, Sun Y, Zhang XP, Lee J, Fu CX, Comes HP. 2009. Molecular phylogeography of East Asian *Kirengeshoma* (Hydrangeaceae) in relation to quaternary climate change and landbridge configurations. The New Phytologist 183:480–495. 19496955 10.1111/j.1469-8137.2009.02876.x

[CIT0059] Rambaut A, Drummond AJ, Xie D, Baele G, Suchard MA. 2018. Posterior summarization in Bayesian phylogenetics using Tracer 1.7. Systematic Biology 67:901–904. 29718447 10.1093/sysbio/syy032 PMC6101584

[CIT0060] Rousset F. 1997. Genetic differentiation and estimation of gene flow from F-statistics under isolation by distance. Genetics 145:1219–1228. 9093870 10.1093/genetics/145.4.1219 PMC1207888

[CIT0061] Rousset F. 2008. genepop’007: a complete re-implementation of the genepop software for Windows and Linux. Molecular Ecology Resources 8:103–106. 21585727 10.1111/j.1471-8286.2007.01931.x

[CIT0062] Sunnucks P. 2000. Efficient genetic markers for population biology. Trends in Ecology & Evolution 15:199–203. 10782134 10.1016/s0169-5347(00)01825-5

[CIT0063] Takezaki N, Nei M, Tamura K. 2010. POPTREE2: software for constructing population trees from allele frequency data and computing other population statistics with Windows interface. Molecular Biology and Evolution 27:747–752. 20022889 10.1093/molbev/msp312 PMC2877541

[CIT0064] Tamaki I, Kawashima N, Setsuko S, Lee JH, Itaya A, Yukitoshi K, Tomaru N. 2019. Population genetic structure and demography of *Magnolia kobus*: variety borealis is not supported genetically. Journal of Plant Research 132:741–758. 31489497 10.1007/s10265-019-01134-6 PMC7196954

[CIT0065] Tang CQ, Dong YF, Herrando-Moraira S, Matsui T, Ohashi H, He LY, Nakao K, Tanaka N, Tomita M, Li XS, Yan HZ, Peng MC, Hu J, Yang RH, Li WJ, Yan K, Hou X, Zhang ZY, López-Pujol J. 2017. Potential effects of climate change on geographic distribution of the Tertiary relict tree species *Davidia involucrata* in China. Scientific Reports 7:43822. 28272437 10.1038/srep43822 PMC5341038

[CIT0066] Van Oosterhout C, Hutchinson WF, Wills DP, Shipley P. 2004. MICRO-CHECKER: software for identifying and correcting genotyping errors in microsatellite data. Molecular Ecology Notes 4:535–538.

[CIT0073] White TJ, Bruns T, Lee S, Taylor J. 1990. Amplification and direct sequencing of fungal ribosomal RNA genes for phylogenetics. In: Innis MA, Gelfand DH, Sninsky JJ, White TJ, eds. PCR protocols: a guide to methods and applications. New York: Academic, 315–322.

[CIT0067] Xie GW. 1997. On phytogeographical affinities of the forest floras between East China and Japan. Chinese Geography Science 7:236–242.

[CIT0068] Xu J, Deng M, Jiang XL, Westwood M, Song YG, Turkington R. 2015. Phylogeography of *Quercus glauca* (Fagaceae), a dominant tree of East Asian subtropical evergreen forests, based on three chloroplast DNA interspace sequences. Tree Genetics & Genomes 11:805.

[CIT0069] Xu B, Wu N, Gao XF, Zhang LB. 2012. Analysis of DNA sequences of six chloroplast and nuclear genes suggests incongruence, introgression, and incomplete lineage sorting in the evolution of *Lespedeza* (Fabaceae). Molecular Phylogenetics and Evolution 62:346–358. 22032991 10.1016/j.ympev.2011.10.007

[CIT0070] Yan SX, Zhang L, Mao RL, Zhu H, Li Y. 2016. Assessment of genetic diversity and population differentiation of *Achyranthes bidentata* (Amaranthaceae) in Dao Di and its surrounding region based on microsatellite markers. Biochemical Systematics and Ecology 69:27–32.

[CIT0071] Yokoyama J, Nakajima M, Nemoto T, Ohashi H. 2000. Preliminary observations on flower visitors of *Lespedeza* subgenus *Macrolespedeza* in Korea. Journal of Japanese Botany 75:248–256.

[CIT0072] Yuan N, Sun Y, Comes HP, Fu CX, Qiu YX. 2014. Understanding population structure and historical demography in a conservation context: population genetics of the endangered *Kirengeshoma palmata* (Hydrangeaceae). American Journal of Botany 101:521–529. 24650862 10.3732/ajb.1400043

