# Supplementary material for: Historical migration and taxonomic entity of Korean endemic shrub Lespedeza maritima (Fabaceae) based on microsatellite loci
Source: AoB Plants. 2021 Feb 18;13(2):plab009. doi: 10.1093/aobpla/plab009 (PMC7983312; doi:10.1093/aobpla/plab009)
Supplement: plab009_suppl_Supplementary_Materials [file plab009_suppl_supplementary_materials.pdf]

Supporting Information

Table S1. Sampling sites of *Lespedeza* species used for comparing genetic structure.

Locality	Coordinate	Code
L. cyrtobotrya		
Isl. Oenarodo, Goheung, Jeonnam, Korea	N 34°26'16" / E 127°30'20.83"	BR
Mt. Daesosan, Yeongdeok, Gyeongbuk, Korea	N36°30'26.03" / E 129°25'35.35"	DS
Mt. Namsan, Gyeongju, Gyeongbuk, Korea	N35°45'36.96" / E 129°12'34.68"	NS
L. maritima		
Isl. Oenarodo, Goheung, Jeonnam, Korea	N 34°26'16" / E 127°30'20.83"	BR
Mt. Gamabong, Namhae, Gyeongnam, Korea	N 34°44'51" / E 128°1'7.62"	GM
Mt. Namsan, Gyeongju, Gyeongbuk, Korea	N35°45'36.96" / E 129°12'34.68"	NS
Mt. Daesosan, Yeongdeok, Gyeongbuk, Korea	N36°30'26.03" / E 129°25'35.35"	DS
L. maximowiczii		
Isl. Oenarodo, Goheung, Jeonnam, Korea	N 34°26'16" / E 127°30'20.83"	BR
Isl. Naenarodo, Goheung, Jeonnam, Korea	N 34°31'12" / E 127°27'16"	NR
Mt. Daesosan, Yeongdeok, Gyeongbuk, Korea	N36°30'26.03" / E 129°25'35.35"	DS
Mt. Namsan, Gyeongju, Gyeongbuk, Korea	N35°45'36.96" / E 129°12'34.68"	NS
L. thunbergii		
Mt. Geumo, Yangsan, Gyeongnam, Korea	N 35°27'06" / E 128°54'55"	GU
Isl. Joyag, Wando, Jeonnam, Korea	N 34°22'44" / E 126°54'48"	JG
Mt. Namsan, Gyeongju, Gyeongbuk, Korea	N35°45'36.96" / E 129°12'34.68"	NS

Table S2. Results of BOTTLENECK testing of 11 populations of *Lespedeza maritima* based on a two-phase (TPM) and stepwise mutation (SMM) models.

Population	Wilcoxon's test		Mode shift
	TPM	SMM	
BG	0.604	0.740	No
CS	0.810	0.912	No
BR	0.455	0.485	No
GM	0.689	0.849	No
YJ	0.455	0.575	No
M	0.339	0.367	No
BS	0.575	0.741	No
GJ	0.912	0.974	No
JY	0.711	0.768	No
NS	0.945	0.968	No
DS	0.995	0.998	No

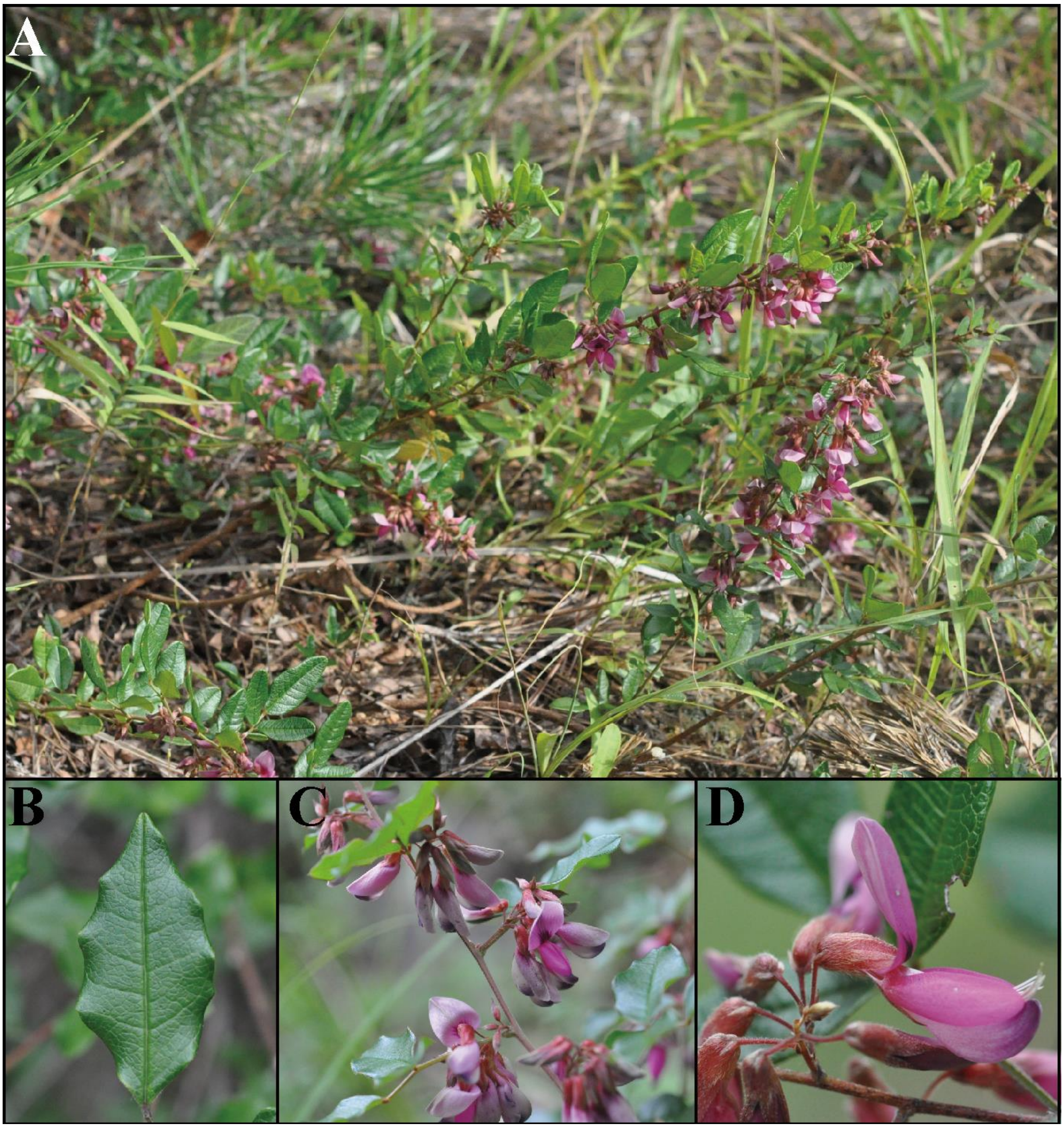

Figure S1. *Lespedeza maritima* Nakai. (A) Habit. (B) Upper surface of leaflet. (C) Inflorescence. (D) Flower.

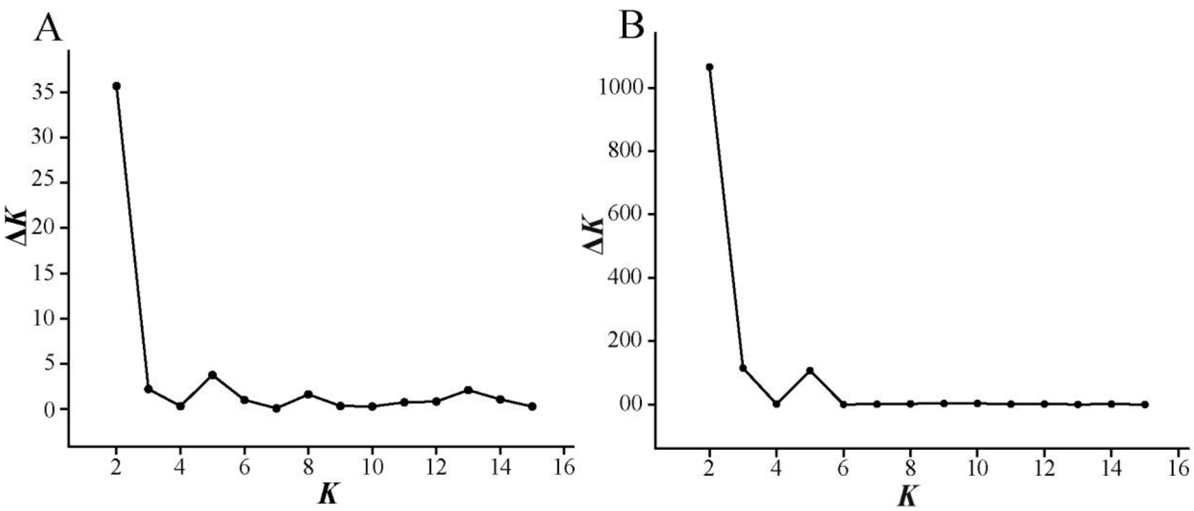

Figure S2. Graph of Δ*K* (y-axis) according to number of clusters (*K*) (x-axis) as calculated based on Bayesian clustering analysis. (A) Result of analysis for 11 populations of *Lespedeza maritima* (Fig. 1). (B) Result of analysis for *L*. *maritima* and related taxa (Fig. 4).

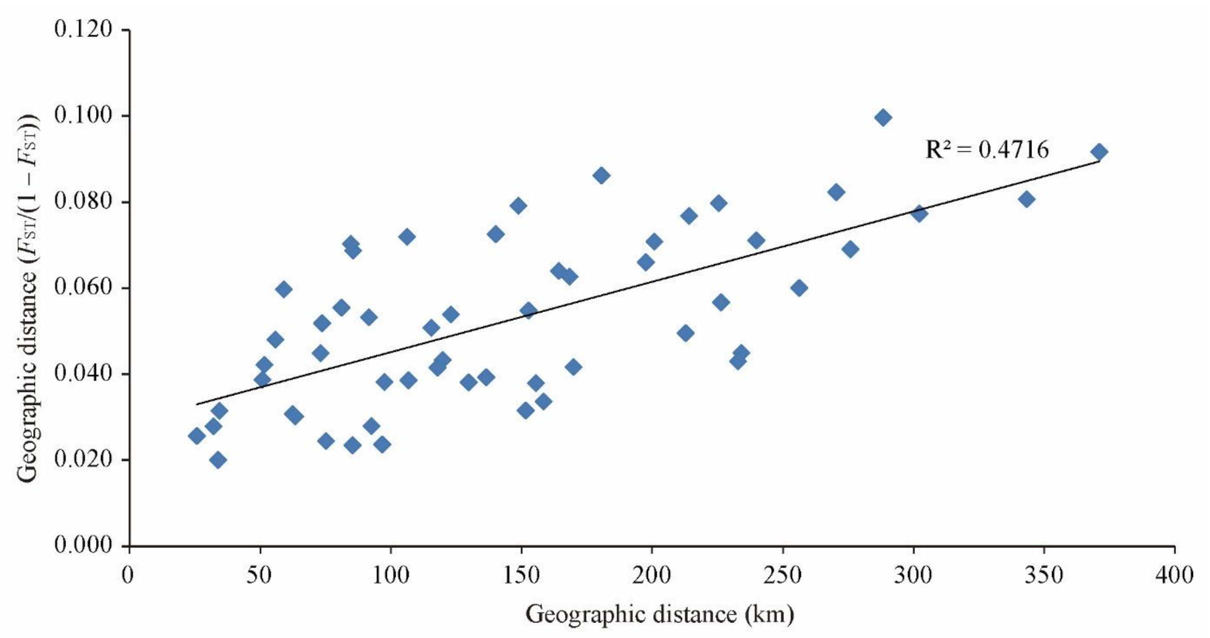

Figure S3. Isolation by distance (IBD) based on relationships between pair-wise genetic distance (F_{ST}/[1 - F_{ST}]) (x-axis) and geographic distance (km) (y-axis) of *Lespedeza maritima* populations. A positive correlation was significant between two distances (R^2 = 0.472; P < 0.001).

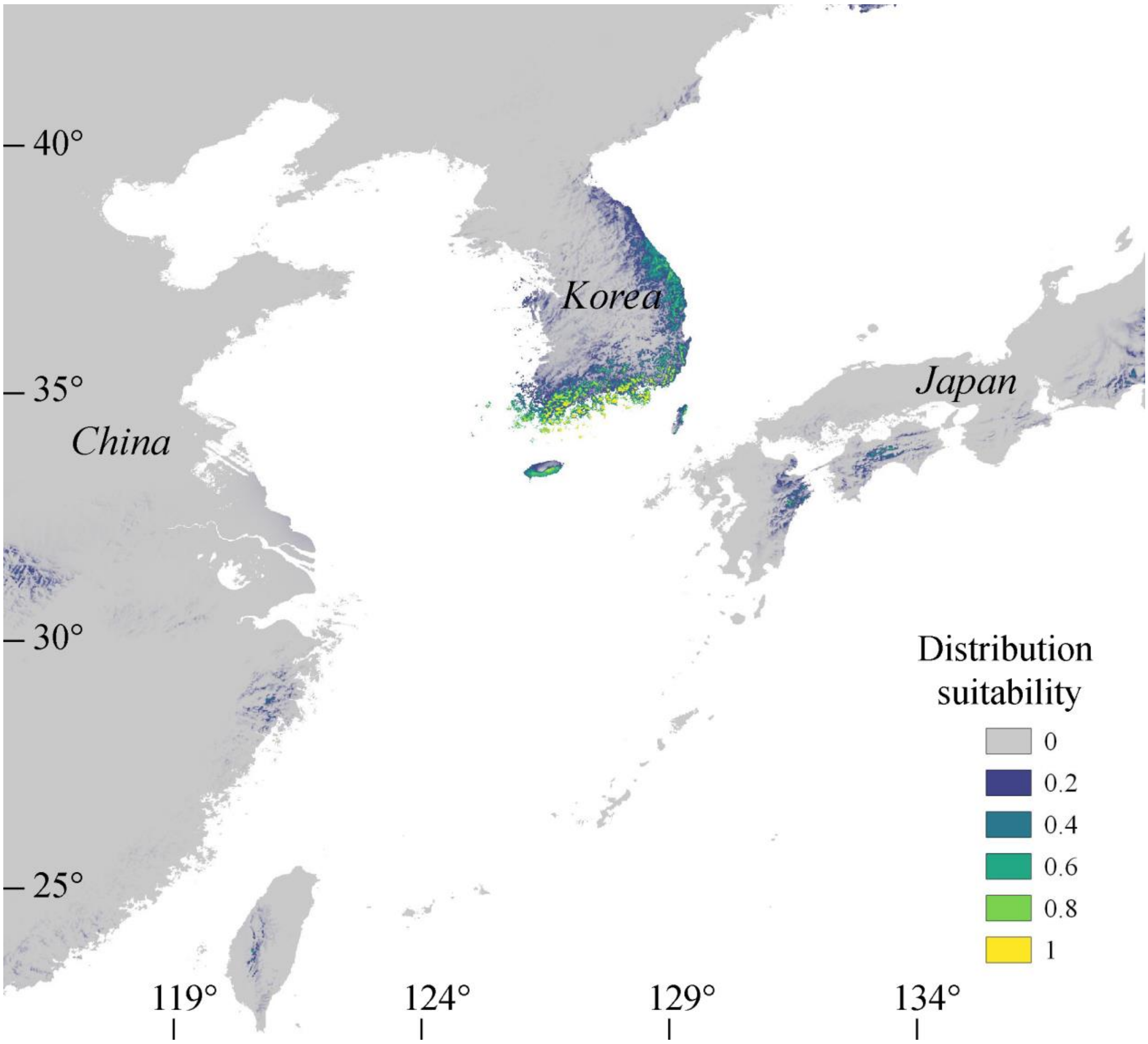

Figure S4. Potential distributions of *Lespedeza maritima* during the current, inferred using ecological niche modelling.

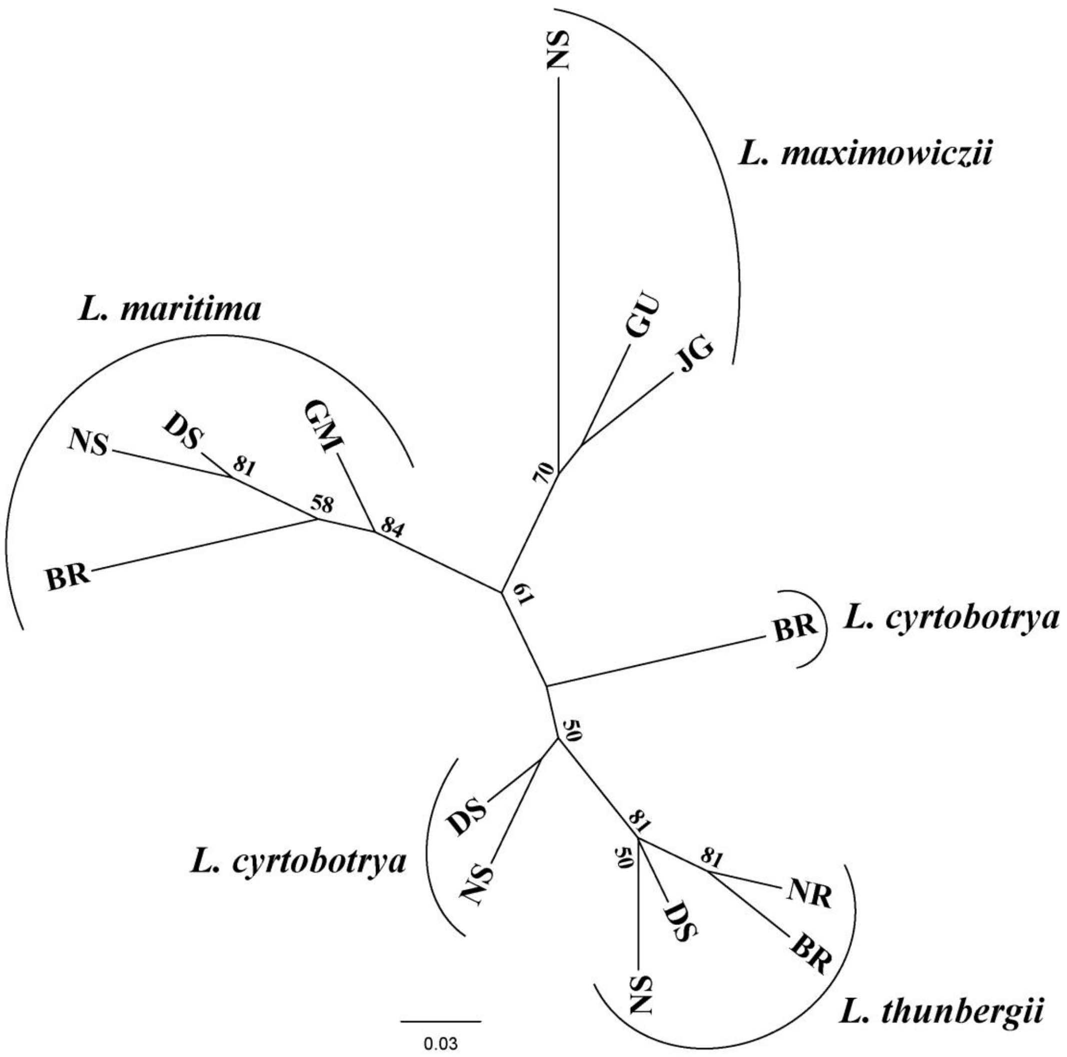

Figure S5. Neighbour-joining (NJ) tree for *Lespedeza maritima* and its related based on eight microsatellite loci. One thousand bootstrap matrices of Nei's genetic distance (D_A) (Nei *et al*. 1983) were implemented. The number of each node indicates the bootstrap value (>50 %).
